# Immigrants and Italian labor market: statistical or taste-based discrimination?

**DOI:** 10.1186/s41118-018-0030-1

**Published:** 2018-02-22

**Authors:** Giovanni Busetta, Maria Gabriella Campolo, Demetrio Panarello

**Affiliations:** 1 Department of Economics, University of Messina, Via Dei Verdi 75, 98122 Messina, Italy; 2 Department of Economic and Legal Studies, Parthenope University of Naples, Via Generale Parisi 13, Naples, 80132 Italy

**Keywords:** Labor market discrimination, First- and second-generation immigrants, Statistical and taste-based discrimination, Experimental economics, Net discrimination

## Abstract

Types of discrimination are usually distinguished by economic theory in statistical and taste-based. Using a correspondence experiment, we analyze which of the two affects Italian labor market the most. In this respect, we studied the difference in discrimination reserved to first- and second-generation immigrants, taking gender differences into account. Even if we want to admit a rational discrimination based on perceived productivity differences (statistical discrimination) against first-generation immigrants (concerning language and education gaps), the same would not be reasonable for second-generation ones. Since they are born and educated in Italy, where they have always lived, the associated discrimination must be taste-based.

## Subject classification codes

C93, J71, J15

## Introduction

According to Arrow (1973), reasons for discrimination can be analyzed from an economic perspective. When a firm discriminates in the hiring process, a positive or negative value is basically assigned to certain characteristics, even if such characteristics are not directly linked to employee productivity.

The US National Research Council defines racial or ethnic discrimination focusing on “differential treatment on the basis of race that disadvantages a racial group, and treatment on the basis of inadequately justified factors other than race that disadvantages a racial group” (Blank et al. 2004). The United Nations define gender discrimination as “any distinction, exclusion or restriction made on the basis of sex which has the effect or purpose of impairing or nullifying the recognition, enjoyment or exercise by women, irrespective of their marital status, on the basis of equality of men and women, of human rights and fundamental freedoms in the political, economic, social, cultural, civil, or any other field” (UN Convention on the Elimination of All Forms of Discrimination against Women—Article 1).

Types of discrimination are usually distinguished by economic theory in statistical and taste-based. The former occurs when the judgment of an individual depends on group characteristics rather than the individuals. The latter occurs, instead, when a group of individuals (either employers or customers) prefer a certain group over another, based on tastes, rather than any economic rationale (see e.g., Lahey 2008 for a review of this literature). The second case includes xenophobia and racism, but also different kinds of personal preferences, the common point being that an employer discriminates against a group of individuals irrespective of the other information he/she has about the candidate (Zschirnt and Ruedin 2016).

In this way, the recruiter does not act in a profit-maximizing manner, but “an avoidance of the psychic cost of contact with the ‘wrong’ race [...] takes precedence” (Riach and Rich 1991, p. 247). Consequently, employers not actualizing racial or gender preferences gain a competitive advantage with respect to the ones actualizing them (Zschirnt and Ruedin 2016).

By contrast, in the case of statistical discrimination, ethnicity acts as a proxy for unobserved information, and members of a specific group are discriminated because employer lacks information (Arrow 1972; Phelps 1972). According to human capital theory, members of minority groups are usually “statistically” characterized by lower human capital compared to majority competitors (Andriessen et al. 2008). This is because they are on average less educated, unfamiliar with host-country institutions and not sufficiently fluent in the language (Zschirnt and Ruedin 2016).

For this reason, even if employers will not always hire the most qualified applicant, statistical discrimination is considered as an acceptable trade-off not to pay the necessary increase in the cost of recruiting to get all the relevant information (Bursell 2007).

While statistical discrimination is usually considered to be efficient in the case of imperfect information (Arrow 1973), taste-based discrimination is inefficient in any case in terms of overall social welfare (Becker 1971).

The aim of this study is to analyze ethnic and gender discrimination in the Italian labor market, distinguishing between their statistical and taste-based components. To measure which part of discrimination is connected to statistical and which to taste-based reasons, we used the difference in discrimination between first- and second-generation candidates.

Italy is particularly relevant as case study because it is a growing immigration country, experiencing for the first time a massive amount of second-generation and Italian-naturalized individuals; the migratory inflow has seen an enormous escalation since 2001 (Fullin and Reyneri 2011), making Italy the second country in Europe for number of immigrants received in the last 20 years (Fullin 2016). We defined first-generation immigrants as the ones who were born abroad and are now living in Italy (all in Rome city center); second-generation immigrants were born in Rome from foreign parents, and they studied in Italy, but they do not have Italian nationality.

Our idea is the following: While first-generation immigrants are individuals who were born and educated in their country of origin, second-generation ones are individuals who were born and educated in Italy. For these reasons, while discrimination against the first generation of immigrants may be driven by reasons related to real differences in productivity (e.g., either linguistic or competence gaps, related to school or university education), the same cannot be said for the second generation (indeed this last category is Italian mother tongue, always living and educated in the country).

Bertrand and Mullainathan (2004), to study ethnic discrimination, used different names distinctly associated with Whites and African Americans. Applicants with White names needed to send about 10 CVs to get one callback, whereas those with African-American names had to send around 15 résumés to get one callback, and this 50% gap in callback rates is statistically significant. Oreopoulos (2011) applied the same methodology in Canada, finding a strong and significant preference for English-sounding names and Canadian education and experience with respect to foreign ones. Similar tests conducted in France (Adida et al. 2010) and in Sweden (Arai et al. 2016) led to the same results. In Italy, one of the first studies in this field was performed by Allasino et al. (2004), who found that most discrimination cases (26.6%) occurred in the first step of the hiring process.

Our study adopts an experimental design that extends the work of the abovementioned authors, introducing the concept of first- and second-generation candidates to decompose overall discrimination into its statistical and taste-based components.

To understand whether the foreign-born individuals are not doing as well as the home-born individuals in the labor market, we collected data through an ad hoc constructed field experiment carried out in Italy in the period between July 2013 and October 2014.

To this extent, we constructed a database sending fictitious CVs to firms advertising real job openings. We did so matching socio-economic background, educational qualifications, work experience, and other characteristics of the applicants, in order to make individuals equivalent in every respect, except for the characteristic which the researcher is investigating (race and sex), and callback rates obtained by applicants can be considered a good proxy of job opportunities.

Indeed, keeping equal all the individual’s characteristics, except for gender and nationality, we can assert that differences in callback rates must necessarily come from discrimination based on such elements.

The Italian National Institute of Statistics reports that 5 million immigrants were living in Italy on January 1, 2015 (8.2% of the population). The four major communities are Romanians (22.6% of all the immigrants), Albanians (9.8%), Moroccans (9.0%), and Chinese (5.3%) (ISTAT 2015a).

Since we wanted to adapt the experimental design to the Italian situation, in our study, we included candidates coming from the four abovementioned most common foreign nationalities of immigrants living in Italy. We decided to use several nationalities instead of just native and immigrants to evaluate whether visible minority groups more distant from natives are discriminated against more than other visible less distant ones (Zschirnt and Ruedin 2016). Germany was also included, as it is the most common nationality in Italy among “rich” western countries (ISTAT 2015a) and should therefore be less discriminated than the candidates coming from poorer and more culturally distant countries.

Being able to develop effective strategies against ethnic discrimination presupposes a detailed analysis of the different types of discrimination (statistical or taste-based) and the subjects involved. “Learning about the ways discrimination affects economic outcomes has the potential to guide policymaking aimed at reducing disparities and it can generate new methods for detecting the presence of discrimination” (Guryan and Charles 2013).

The rest of the paper is organized as follows: In the “Theoretical background” section, we provide a short review of the literature. The experimental design is described in the “Experimental design” section. The “Descriptive results” section shows the descriptive results, and the “Empirical analysis” section shows the empirical results of our models. Finally, we conclude our study in the “Conclusions” section.

## Theoretical background

### Statistical and taste-based discrimination

The first studies investigating ethnic discrimination in the labor market consisted of audits made in the USA, where people from different ethnic groups (White, Hispanics, and Afro-Americans) were asked to apply for job offers and to attend job interviews (Newman 1978; McIntyre et al. 1980; Heckman and Siegelman 1992; Heckman, 1998; Altonji and Blank, 1999).

Research conducted by the “International Labour Office (ILO)” shows that most of the discrimination found in hiring process occurred at the starting point of the recruitment when the candidate’s CV is examined. Riach and Rich (2002) in a comparative study found significant ethnic discrimination in the initial stage (nearly 90% of the total level of discrimination) in all the countries examined. For this reason, we decided to analyze the first step of the hiring process. The idea is that if discrimination is going to occur, it is easier for firms not to interview a potential employee rather than firing him/her.

Having established the existence of ethnic discrimination in the first stage of the hiring process, several studies concentrated in evaluating how to split the total amount of such discrimination within its statistical and taste-based components.

To do so, we decided to adapt the approach conducted by some American studies (Bertrand and Mullainathan 2004; Oreopoulos 2011) to the Italian labor market, building up an ad hoc field experiment.

In terms of ethnic hierarchies, results of the main studies show several different patterns depending on the country analyzed. While the discrimination in Austria is highest for Arabs, followed by Chinese, Indians, Pakistani, and Bangladeshi (Hofer et al. 2013), the results for Australia show the minimum level of discrimination for Italian immigrants followed by Aborigine candidates, Middle Eastern candidates, and Chinese (Booth et al. 2012), and Ireland Asian immigrants are less discriminated than Germans and Africans (McGinnity and Lunn 2011).

Even if the most discriminated ethnicities depend on the country analyzed and, consequently, on cultural reasons, results show a clear ethnic hierarchy, which is robust to differences in skill levels and suggests, in this way, a relevant amount of taste-based discrimination (Zschirnt and Ruedin 2016).

Concerning European Union, an issue of ethnic discrimination appeared on the European political agenda. The consequent adoption of two new directives (Directives 2000/43/EC and 2000/78/EC) brought to an increase in the level of discrimination, related to its taste-based component.

Several studies (Kaas and Manger 2012; Schneider et al. 2014; Weichselbaumer 2015) analyzing Germany show the presence of lower levels of discrimination in this country than elsewhere. As this country is characterized by CVs requiring more detailed information which reduce informational asymmetry, the results of a lower level of discrimination reveal a reduced level of the statistical component. Deeply, on the one hand, these results suggest that statistical discrimination plays a relevant role; on the other hand, as discrimination is still relevant even if the level of information is huge, the results reveal a massive amount of taste-based discrimination.

### First- and second-generation immigrants: international and Italian context

Some of the studies on this topic used first- and second-generation immigrants to make a difference between the two reasons for discriminating. Even if not all of them agree on how to define first- and second-generation individuals, most of the studies explicitly mention that they both have been schooled in the country analyzed.

There is no general evidence of a lower discrimination against second-generation candidates, suggesting the existence of a strong taste-based component. In the multivariate models, the coefficient for second generation appears to be negative, implying the existence of statistical discrimination (see, e.g., Zschirnt and Ruedin (2016) for a review of the main literature).

According to Zschirnt and Ruedin (2016) “Second-generation immigrants are thus likely to be perceived more positively, with generation serving as a signal of civic integration.”

The employment opportunities for children of immigrants are a matter of growing concern (Crul and Vermeulen 2003; Heath et al. 2008; Thomson and Crul 2007). Even if they have usually acquired linguistic and competence skills comparable to children of natives and they have therefore substantial capacity for labor market integration (Alba and Waters 2011, Midtbøen 2016), they do not often have comparable working opportunities (Crul et al. 2012; Heath and Cheung 2007; OECD 2010). Second-generation immigrants face different disadvantages depending on the country in which they live. In countries such as Austria, Belgium, and Germany, they experience these disadvantages even after getting employed. In countries such as Britain, Sweden, and Norway, they face barriers only at the entrance to the labor market (Heath et al. 2008; Hermansen 2013; Midtbøen 2016).

Looking at Italian labor market, the level of immigrants’ integration still appears to be very low in terms of employment and qualified occupations (Fullin and Reyneri 2011).

According to the survey on “Integration of the second-generation (ISG)” carried out by ISTAT in 2015 and involving lower and upper secondary schools, immigrants born in Italy perform almost the same scores as Italian students, meaning that there is no significant statistical difference between the two categories. Nowadays, immigration in this respect appears to be different from old European immigration, not being anymore poor education of immigrants the main reason for their higher difficulties in finding a job. Indeed, they do not find challenging difficulties in accessing unskilled and semi-skilled manual jobs, as they experience in self-employment and non-manual jobs (Fullin and Reyneri 2011).

Moreover, teachers declared in general a good level of integration of foreign students, who feel to be Italian citizens often more than citizens of their country of origin. Moreover, in our sample, Italian and immigrant candidates are identical in every respect because their CVs contain equivalent information. Consequently, whatever is the mean statistical performance of the categories, in our experiment, Italian and immigrant candidates are identical.

Furthermore, “not to be Italian born represents a huge difficulty in finding a job in Italy (especially an appropriate one) for 36.2% of foreigners and 22% Italian naturalized” (ISTAT 2015b). The sample collected by ISTAT belongs to Quarterly Labour Force Survey, and the results of the analysis locate in poor knowledge of Italian language, in the non-recognition of qualifications from abroad and in socio-cultural reasons the main difficulties to enter the labor market. On the one hand, this analysis considered the point of view of immigrants or naturalized and their subjective perception about the reasons determining difficulties in obtaining a job. On the other hand, it tells us nothing concerning the real motivations underlying firm’s hiring choices. For this reason, instead of analyzing immigrants’ perception, we preferred to look at the firm’s behavior and, indirectly, at the reasons motivating their choice not to hire foreigners and naturalized candidates.

### Double segregation: international and Italian context

We also include gender in our analysis, taking into consideration gender differences concerning each ethnicity separately. Women are, on average, less likely to be employed than men, and important differences persist between men and women in the labor market (Boeri et al. 2005). The connected discrimination also exists when we deal with immigrants, often becoming, in that case, even more pronounced (Altonji and Blank 1999). This kind of discrimination is referred as by literature in terms of double discrimination: women are indeed discriminated twice, both because they are women and immigrants (Boyd 1975).

In terms of double discrimination in the Italian labor market between migrant men and women and to the ethnic wage gap between Italian and migrant women, Piazzalunga (2015) found that the second is much larger than the first one, confirming that immigrant women face a double discrimination based on both ethnicity and gender.

## Experimental design

For this study, we collected data through a field experiment carried out in Italy in the period between July 2013 and October 2014. During that period, we sent 22,000 fictitious résumés, answering to 1000 real online job postings. A large sample size like this permits us to have a sufficient statistical power to investigate not only the difference between Italians and immigrants (of first and second generation) but also different ethnic groups. We implemented empirical evidence, such as Booth et al. (2012), using at the same time different ethnicities and first- and second-generation.

To control the characteristics of the CVs, we produced fictitious résumés based on the European Europass format and structure creating the identities as follows: one Italian man, five first-generation immigrant men, five second-generation immigrant men, one Italian woman, five first-generation immigrant women, and five second-generation immigrant women. Immigrants were equally distributed and came from Romania, Albania, Morocco, People’s Republic of China, and Germany.

Among these nationalities, Germans and Romanians are European Union citizens, who do not need a visa to live and work in Italy. Albania and Romania are usually considered very similar nations since they have many commercial contacts with Italy and they were socialist republics until the 1990s. For this reason, their comparison is useful to show whether being part of the European Union makes a significant difference in the probability of being hired.

For each offer, identities are constructed with equivalent skills (i.e., in terms of the level of education and working experience) and information, to be perfectly comparable differing only by name, surname, gender, city of birth, postal address, and e-mail address.

Each identity was assigned its own name, surname, gender, city of birth, postal address in Rome city center, and e-mail address. About physical features, we chose not to include any pictures in the résumés to avoid further discrimination based on the candidates’ appearance; in this way, employers do not have any information about skin color and physical distance from Western features in advance, being only able to make subjective assumptions. Moreover, we did not include any telephone numbers, as we did not have real respondents able to answer the calls.

The search engines from which the job offers were extracted are subito, monster, kijiji, miojob, combinazioni s.r.l., free work, lavoro.corriere, job365, and vivastreet. According to the position in Google search results, these are the most used job search engines in Italy.

Unlike the procedure applied by Rooth (2009), we did not change associations between names, nationalities, and institutions attended in our experiment. Thus, we maintained credibility in the application, since we could not exclude that the same vacancy was present in several of the web search engines posting job offers. Moreover, in order to reduce the probability of applying to the same job offer more than once, following the methodology used in studies such as Pager et al. (2009), we drew job offers from the main search engines, taking a simple random sample of advertisements each week. In this way, even if we want to admit the possibility of some kind of distortion, produced by applying for the same firm more than once, it will not affect the preference for different candidates.^1^

We used the most common names for each nationality to minimize the concerning bias, avoiding in this way misattribution to other ethnic groups, or connotations of class or socio-economic status (Zschirnt and Ruedin 2016).

In terms of years, different candidates to the same offer all have the same age. Deeply, they are all 19 years old if the offer requires a high school diploma, 24 if it requires graduation, and one additional year for each year of working experience required by the offer. This methodology ensures that perceived productivity characteristics on the supply side are held constant.

All the candidates specified their language knowledge according to the Common European Framework of Reference for Languages, which is the standard evaluation method in the Europass CV format that we used. All the first-generation candidates specified their country’s language as mother tongue, a C2 (highest level) knowledge of Italian, and knowledge of other languages according to the job offer’s requirements. Second-generation candidates specified Italian as their mother tongue and a B2 (post-intermediate level) knowledge of their country’s language; in this case as well, other languages were added according to job offers’ requirements. Italians, on the other hand, specified in their résumés to have Italian as their mother tongue, while additional languages were included only when requested by the job announces.

The last characteristics that we considered in the analysis are the qualifications required, dividing job openings into offers requiring graduation, high school diploma, and no qualification at all. We chose comparable institutions; while we chose the most important schools and universities of their own country for first-generation immigrants, we chose same Italian schools and universities for Italian and second-generation candidates. Finally, to avoid matching problems and to be competitive with respect to other applicants, for each different job offer, we added the characteristics, i.e., education, work experience, language, and computer skills, which completely fulfill the skills required by the firms to each association already made.

Our design strategy of sending fictitious CVs, which exactly meet the firms’ requirements, allowed us to eliminate matching problems as a possible explanation for the difference in the rate of response. Moreover, we classified all job openings according to whether the position involves face-to-face contact with the public. We classified as front office those job postings which either explicitly stated that the job requires face-to-face contact with people, or where such contact could be unequivocally inferred from the job advertisement. Otherwise, the job is classified as back office. We included in the first category, for instance, jobs belonging to fields like sales and customer service. By contrast, in the back office category, we decided to include jobs like accounts management, budgeting, industrial engineering, and computer programming. In this regard, it must be noted that in the last 15 years, an increase in foreign employees working in contact with the public was observed in Italy (Allasino et al. 2004).

The degree of differential treatment was quantified by using the difference between groups in the number of callbacks for a job interview. Responses were classified as callbacks if the employer requested an applicant to contact them (not just for clarification) or invited him/her for an interview. To minimize inconvenience to the employer, invitations were promptly declined as employers who contacted an applicant were contacted via email and told that the applicant had accepted another position and was no longer looking for employment.

## Descriptive results

After receiving the CVs, firms can react in one of the following ways: both applicants are invited to an interview, only the majority/native applicant is invited to an interview, only the minority/immigrant applicant is invited, and none of them are invited. In the last case, we can consider the no callbacks either as a case of equal treatment, because all the candidates are treated symmetrically (Cross et al. 1990), or as a non-observation. In this respect, there are, indeed, many reasons why applicants might be rejected before the employer even considers their ethnic background (Riach and Rich 2002). Since we cannot control for the reason why some firms do not answer to any of the candidates, we decided to follow the second approach that considers the no callback cases as non-observations (Riach and Rich 2002; Kaas and Manger 2012), restricting the sample to the ones that have replied to at least one CV (n. 229 companies, subjects 5038). As stated by Riach and Rich (2002): “There are many reasons why job applicants may be rejected before an employer has to confront race (or sex). Initial screening may be based on timing of applications, age, current employment status, etc.” Indeed, we are concerned to better interpret the real opportunities of the labor market. In this way, the sample is composed of 458 Italian men and women, 2290 first-generation immigrants, and 2290 second-generation ones.

Table 1 shows the descriptive statistics for all variables of the total and restricted sample. It is also stratified by ethnicity and shows the callback rate.

**Table 1:** Descriptive statistics—percentages of all variables by nationality considering the full sample, the restricted sample, and the callback rate

	Italian			First generation			Second generation		
	Full sample n. 2000	Restricted sample n. 458	Callback rate n. 237	Full sample n. 10,000	Restricted sample n. 2290	Callback rate n. 539	Full sample n. 10,000	Restricted sample n. 2290	Callback rate n. 734
Woman	0.50	0.50	0.39	0.50	0.50	0.37	0.50	0.50	0.37
Graduate	0.24	0.24	0.27	0.24	0.24	0.26	0.24	0.24	0.28
High school	0.43	0.43	0.43	0.43	0.43	0.42	0.43	0.43	0.46
No title	0.33	0.33	0.30	0.33	0.33	0.33	0.33	0.33	0.26
Front office	0.46	0.44	0.45	0.46	0.44	0.33	0.46	0.44	0.42
North-center	0.77	0.85	0.84	0.77	0.85	0.89	0.77	0.85	0.84

**Fig. 1:**
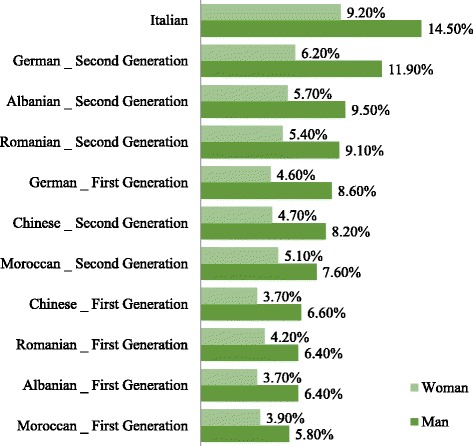
Callback rates by nationality, generation, and sex

The preliminary descriptive analysis about the total sample (see Fig. 1) shows that a relevant difference exists in the treatment reserved to Italians compared to immigrants in the labor market, consistently with the results indicated in the annual report on the presence of migrants in Italy (Ministry of Labour and Social Policy 2016). Indeed, Italian applicants received a much higher proportion of replies than immigrants (Table 2) also considering the applicants’ gender (Table 3). Analyzing the callback rates obtained by immigrants more in detail, another difference appears, concerning whether the candidate is a first- or a second-generation one. Callback rates are lower for first-generation immigrants than for second-generation ones. Nevertheless, second-generation candidates maintain a significant level of discrimination too, compared to Italians. This result is consistent with the ones shown by Georgiana and Agafiţei (2016). Even though most second-generation immigrants feel to be Italian citizens in every respect (in line with “Integration of the second-generation” carried out by ISTAT 2016), they continue to face more difficult conditions in the labor market than their Italian counterparts (in line with OECD 2014).

**Table 2:** Callback rates for the full sample and the restricted sample

	Positive responses	Full sample		Restricted sample	
	*N*	CVs sent	Callback rate (%)	*N*	Callback rate (%)
Italian	237	2000	0.12	458	0.52
First generation	539	10,000	0.05	2290	0.24
Second generation	734	10,000	0.07	2290	0.32

The restricted sample is formed by 458 Italian men and women, 2290 first-generation immigrants and 2290 second-generation ones

**Table 3:** Callback rates by ethnicity and gender

	Restricted sample	Woman		Man	
	*N*	*N*	%	*N*	%
Italian	229	92	0.40	145	0.63
First generation	1145	201	0.18	338	0.30
Second generation	1145	271	0.24	463	0.40

In the restricted sample—total Italian candidates = 458; first generation = 2290; second generation = 2290

The preliminary results of our experiment show the existence of a relevant difference in the treatment reserved to Italians and immigrants (Table 3). Indeed, while 52% of Italian applicants received a reply, only 28% of immigrants obtained it. Significant differences also emerge in terms of gender gap. Generally, female candidates obtain massively lower callback rates than men. Considering the potential gender gap within each subgroup (Italian, first-, and second-generation), the table shows that a difference in callback rate also exists between women and men. Among the female Italian candidates, only 40% receive a positive response (men 63%), while among first-generation women, only 18% (30% if men). Finally, among second-generation female immigrants, the percentage of callback is equal to 24% (40% if men). These preliminary results confirm that, in general, women are the most discriminated category in the Italian labor market compared to men in each subgroup.

In terms of nationalities, first- and second-generation Moroccan and Chinese are the most discriminated in our database. This last result is consistent with the main literature which explains ethnic hierarchy as an indicator of a relevant amount of taste-based discrimination (Zschirnt and Ruedin 2016).

Moreover, Moroccan candidates, as they come from a Muslim country, could be even more discriminated for religious reasons connected to Islamophobia.^2^

Conversely, applicants coming from Germany, both first and second generation, seem to be the preferred immigrant ethnicity overall. According to Zschirnt and Ruedin (2016), “taking taste-based discrimination seriously, it can be assumed that more distant and visible minority groups are discriminated against more than other groups.”

Even if callback rates associated to women are systematically smaller than the ones associated to men, this result is not surprising considering the strong gender gap characterizing the Italian labor market (Anxo et al. 2011; Campolo and Di Pino 2012; Busetta and Fiorillo 2016). It is also interesting to note that gender gap between Italians is often higher than the gap between Italians and immigrants of the same sex. In other words, discrimination against women with respect to men is higher than discrimination against immigrants with respect to Italians. For instance, callback rates for Italian women are lower than the ones obtained by German and Albanian men. This result is not dissimilar from the one reached by the major literature on this topic. Stereotypes and media images of immigrants tend to be less relevant within women than men (Bovenkerk 1992; Andriessen et al. 2012). “This may lead to women being perceived as better integrated into and less threatening to society than immigrant men, and thus lower discrimination for women” (Zschirnt and Ruedin 2016).

Even after restricting the sample, callback rates associated to second-generation immigrants are quite smaller than the ones associated to Italians. This last result shows that discrimination in the labor market is relevant also for second-generation immigrants. Splitting immigrants by generation, we conducted our study considering two potential kinds of discrimination: statistical and taste-based.

Following the most relevant literature on the topic and to investigate this last relation, we decided to perform a correspondence test to our sample such as in Riach and Rich (2002) and Drydakis (2009) among others.

**Fig. 2:**
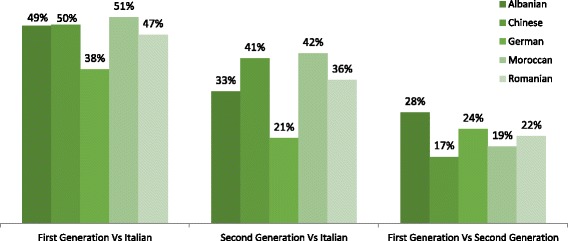
Aggregate correspondence test—net discrimination (%) by ethnic origin

Therefore, we calculated the net discrimination between Italian and first-generation candidates to observe the maximum amount of discrimination reserved to immigrants (Fig. 2); indeed, we split such a discrimination into the one between Italian and second-generation and the one between second- and first-generation candidates. While the last discrimination is between two categories which can differ in terms of education and skills, the same cannot be said for the first one (between Italian and second-generation candidates). Consequently, while the first case could pertain to statistical discrimination, the second one must necessarily be taste-based.

In this respect, we followed the results obtained by ISTAT (2016). According to this study, immigrant students are not systematically less prepared than Italian ones both in terms of language knowledge and general skills.

Using the correspondence test, two applications sent to real job offers are similar, except for the single characteristic that is to be tested. In this way, analyzing callbacks, it is possible to identify unequal treatment based on the unique different characteristic that, in our case, is the ethnic provenience. In Table 4, we reported the results of the correspondence test, considering second generation as minority group and Italians as majority one. For this reason, we calculated callback rates by education level (*graduate*, *high school*) and contacts with customers (*front office*).

**Table 4:** Aggregate correspondence test results (second-generation vs Italian applicants) by education level and contacts with customers

	Jobs	No one invited	At least one invited	Equal treatment	Discrimination against		Net discrimination		Relative callback rate	*χ* ^2^ test
			1	2	Second-generation 3	Italians 4	3 − 4	$\frac{3-4}{1}$	$\frac{\left[\left(2+3\right)/1\right]\ }{\left[\left(2+4\right)/1\right]}$	
Nationality	No.	No.	No.	No.	No.	No.	No.	%		*p*
Callback: graduate										
Albanian	110	40	70	36	29	5	24	34.29	1.59	***
Chinese	110	40	70	29	36	5	31	44.29	1.91	***
German	110	38	72	48	17	7	10	13.89	1.18	***
Moroccan	110	41	69	28	37	4	33	47.83	2.03	***
Romanian	110	42	68	38	27	3	24	35.29	1.59	***
Callback: high school										
Albanian	196	83	113	58	44	11	33	29.2	1.48	***
Chinese	196	80	116	49	53	14	39	33.62	1.62	***
German	196	83	113	69	33	11	22	19.47	1.28	***
Moroccan	196	81	115	46	56	13	43	37.39	1.73	***
Romanian	196	83	113	55	47	11	36	31.86	1.55	***
Callback: front office										
Albanian	200	83	117	54	52	11	41	35.04	1.63	***
Chinese	200	81	119	37	69	13	56	47.06	2.12	***
German	200	79	121	64	42	15	27	22.31	1.34	***
Moroccan	200	82	118	41	65	12	53	44.92	2.00	***
Romanian	200	84	116	53	53	10	43	37.07	1.68	***

The first column provides the ethnic origin of the applicant, the second one shows the number of applications sent, the third one shows the cases in which no individuals were invited for an interview, column four shows the cases in which at least one applicant was invited, column five shows the cases in which both were invited, column six shows the cases in which only the Italian candidates were invited, and the seventh one shows the cases in which only the second-generation candidates were invited. In the following two columns, we calculated the net discrimination against second-generation immigrants, and, finally, in the last columns, we reported the *p* value related to the chi-squared test. Chi-squared tests were conducted on the callback rates, and the results are indicated as *** significant at the 0.001 level. A positive sign indicates discrimination against the minority applicant (second-generation candidates). The null hypothesis is that discrimination against the second generation is equal to discrimination against Italians, therefore both individuals are treated unfavorably equally often

Net discrimination = (discrimination against second-generation—discrimination against Italians)/(at least one invited); Relative callback rate = success of majority/success of minority = [(2 + 3)/1]/[(2 + 4)/1]

Using the correspondence test, we also calculate the net discrimination in callbacks between first generation and Italians, second generation and Italians, and finally, between first and second generation by ethnic origin. For lack of brevity, in Fig. 2, we show the percentage of net discrimination calculated.

As we could imagine, the highest level of discrimination is the one between first-generation immigrants and Italian candidates. Moreover, results reveal a relevant discrimination also between first- and second-generation candidates. Both previous discriminations could be connected to educational and language gaps (statistical discrimination). The impressive result is the one between second-generation candidates and Italians. As the two categories are both born and always living and educated in Italy, this last discrimination can only be derived by reasons connected to racism (taste-based discrimination).

In this respect (second diagram in Fig. 2), we have observed a level of net discrimination between second-generation and Italian candidates higher than 30% compared to Italians for all our categories of ethnicity, except for Germans.

Starting from these preliminary results, we concentrated our analysis on the relation between taste-based discrimination and education (*graduate* and *high school*) and taste-based discrimination and *front office*, considering the impact of education and the level of *vìs a vìs* contact required by the job offer (see Table 4).

The concerning results show that the different education levels required by the job increase or decrease these net discriminations. In general, we observed that applying for a job requiring graduation increases the net discrimination for Albanians (from 33 to 34%), Chinese (from 41 to 44%), and Moroccans (from 42 to 48%). Only for Germans and Romanians we observe a positive effect of the graduation (from 21 to 14% and from 36 to 35%, respectively). The opposite occurs if we consider high school diploma as the level of education required (Albanians 29%, Chinese 33%, Moroccans 37%, Romanians 31%). For both first- and second-generation German candidates, net discrimination decreases for jobs requiring a high school diploma, but the associated decrease is less pronounced in this last case (up to 19%) than in the ones for which graduation is required (up to 14%). Finally, for jobs entailing contacts with customers, we can observe that the net discrimination increases for all second-generation immigrants (Albanians from 33 to 35%, Chinese from 41 to 47%, Germans from 21 to 22%, Moroccans from 42 to 45%, Romanians from 36 to 37%).

## Empirical analysis

In this section, we analyze the effect of ethnicity on the probability of being called back for an interview. To capture the effect of discrimination, we estimated this probability (callback) in different Probit models:

1 $$ {Y}_i^{\ast }=\alpha +{\beta}_1{\mathrm{italian}}_i+{\beta}_2\mathrm{north}-{\mathrm{center}}_i+{\beta}_3{\mathrm{woman}}_i+{\beta}_4{\mathrm{graduate}}_i+{\beta}_5{\mathrm{high}\ \mathrm{school}}_i+{\beta}_6{\mathrm{front}\ \mathrm{office}}_i+{\varepsilon}_i $$

where Y^*^ is the latent variable reflecting the probability of the *i*th candidate of receiving a callback from a firm. If Y^*^ > 0, the corresponding observed dummy variable, *Y*, is equal to 1 (Callback—yes = 1). If Y^*^ = 0, the corresponding value of the dummy variable, *Y*, is equal to 0 (Callback—no = 0). On the right side of the equation, Greek letters refer to parameters. Namely, *α* is the constant term, Italian is a dummy variable and it refers to the ethnicity of the candidate (1 = the candidate is Italian; 0 = otherwise); *ε* is the disturbance term.

The main explanatory variables are the firm’s geographical area (north-center—1 = yes; 0 = otherwise), the sex of the candidate (woman—1 = yes; 0 = man), the education level required by the firm and specified in the CVs (graduate—1 = yes, 0 = otherwise; high school—1 = yes, 0 = otherwise; reference—no title), if the job offer requires contacts with customers (front office—1 = yes, 0 = back office). If ${\widehat{\beta}}_1=0$, Italians and immigrants had the same probability of obtaining an interview. It means that being an Italian does not either reduce or increase the probability of obtaining a job interview. While if ${\widehat{\beta}}_1<0$, immigrants had a higher probability than Italians of obtaining a job interview; if ${\widehat{\beta}}_1>0$, they had a lower probability than Italians of obtaining it.

The objective of our analysis is to analyze whether discrimination based on ethnic origin affects candidate’s opportunity of obtaining a job interview in the Italian labor market. Therefore, for completeness, we replicate our model considering different subsamples, estimating four identical Probit models applied to different subsamples. Model 2 (Italian) contains only observations on the Italian candidates, and in model 3 (immigrant), only observations on the immigrant ones. Finally, in the last two models (model 4 and model 5), we split immigrants into two subsamples, first and second generation.

In Table 5, we show the results of our Probit model and the relative marginal effects.

**Table 5:** Estimation results of Probit models and marginal effects

	Model 1	Model 2		Model 3		Model 4		Model 5	
	All	Italian		Immigrant		First generation		Second generation	
	Coeff.	Coeff.		Coeff.		Coeff.		Coeff.	
Italian	0.65*** (0.22***)								
Woman	− 0.45*** (− 0.15***)	− 0.59*** (− 0.23***)		− 0.43*** (− 0.14***)		− 0.39*** (− 0.12***)		− 0.47*** (− 0.17***)	
Graduate	0.23*** (0.08***)	0.34* (0.13*)		0.22*** (0.07***)		0.12 (0.04)		0.32*** (0.11***)	
High school	0.15*** (0.05***)	0.15 (0.06)		0.15** (0.05***)		0.03 (0.01)		0.26*** (0.09***)	
Front office	− 0.16*** (− 0.05***)	0.05 (0.02)		− 0.18*** (− 0.06***)		− 0.34*** (− 0.10***)		− 0.06 (− 0.02)	
North-center	0.07 (0.02)	− 0.02 (− 0.11)		0.08 (0.03)		0.25** (0.07***)		− 0.05 (− 0.02)	
Constant	0.49***	0.20		− 0.50***		− 0.66***		− 0.37***	
Pseudo *R*^2^	0.046	0.047		0.029		0.035		0.035	
Log likelihood	− 2933.84	− 302.38		− 2628.38		− 1205.86		− 1386.75	
AIC	5881.68	616.77		5268.75		2423.73		2785.50	
BIC	5927.35	641.53		5307.33		2458.14		2819.92	

Marginal effects in brackets

*p < 0.05; **p < 0.01; ***p < 0.001;

To analyze the probability of obtaining a callback, we first considered a model which includes all the categories and a dummy variable Italians as regressor (model 1). In the other models (models 2–5), we considered the subsample of Italians, immigrants, first-, and second-generation candidates. Model 1 shows that the probability of obtaining a callback increases if the candidate is Italian. The results of this analysis confirm the ones reported above. In general, the coefficients maintain same sign and statistical significance in all the five models, confirming the robustness of our results. For this reason, we analyzed all the models together. In terms of gender gap, the coefficients associated to the variable woman reveal a strong level of statistically significant discrimination for both Italian and immigrant women. Gender gap seems to be smaller within immigrants than within Italians, especially if we compare Italians with first-generation immigrants. The education level (*graduate* and *high school*) plays an important role (positive and significant coefficients), for all candidates. The interesting result is that the increase in probability is higher for Italians than for immigrants but becomes not significant for first-generation immigrants. For this kind of candidate, a higher level of education does not necessarily involve a greater probability to obtain a job interview. This result is supported by the main literature on the topic (Heath and Cheung 2007 and, for Italy, Fullin and Reyneri 2011). Therefore, we could argue that the reason underlying this kind of discrimination concerns the unequal perception of recruiters about graduation obtained in foreign countries, compared to graduation obtained in Italy.

If we analyze results concerning jobs requiring contacts with customers (*front office*), the associated coefficient is negative and significant for the model including all the immigrants and for the one including only first-generation immigrants. This last result indicates statistical discrimination because it seems that firms consider only first-generation immigrants less skilled, most probably in terms of language knowledge, than Italians and second-generation candidates.

Finally, the coefficients associated to the firm’s geographical area (*north-center*) show that it is easier for immigrants to obtain a job interview if they apply for a firm located in the north-center, but this result is statistically significant only for the subsample of first-generation candidates.

## Conclusions

The aim of this paper is to analyze ethnic and gender discrimination in the Italian labor market, using first- and second-generation candidates to distinguish between statistical and taste-based discrimination. To do so, we build up an ad hoc created database sending fictitious CVs to firms advertising real job openings.

We found a first significant discrimination against women which interacts differently depending on the candidate (Italian, first-, or second-generation immigrant). In this respect, even if callback rates associated to women are systematically smaller than the ones associated to men, gender gap between Italians is often higher than the one between both first- and second-generation immigrants. As we have shown in Table 5, Italian women suffer a higher reduction (− 23%) than immigrant women in the probability of obtaining a job interview compared to their male counterparts. In this respect, first-generation immigrant women obtain a reduction of 12% compared to their male counterparts, and second-generation immigrant women obtain a reduction of 17% with respect to their male counterparts. This first result seems to be particularly relevant, and we are thinking, for this reason, to use it as starting point for a new research question.

The highest level of discrimination is the one between first-generation immigrants and Italian candidates. It can be divided into two parts: the one between first- and second-generation candidates and the one between second-generation and natives. While the first one is between candidates which are different in terms of professional skills and level of Italian language knowledge and can therefore be connected to statistical reasons, the second one affects candidates with the same level of professional skills and language knowledge, and it can only be addressed to taste-based reasons.

Even if results reveal a relevant discrimination also between first- and second-generation candidates (statistical discrimination), the most remarkable result is the one between second-generation candidates and Italians; as the two categories are both born, always living, and educated in Italy, this last discrimination must necessarily be taste-based, which is always inefficient in terms of overall social welfare.

Starting from these preliminary results, we concentrated our analysis on the relationship between discrimination and education and discrimination and level of *vìs a vìs* contact required by the job offer. The interesting result is the following: a higher education required by the job offer increases the probability of being called back more for Italians than for immigrants, and this increase completely disappears for first-generation candidates.

As graduation is to be considered equivalent by law—at least the ones obtained in the European Union—this last form of discrimination must be a taste-based one. This result is in line with the one obtained by Fullin and Reyneri (2011). According to the authors, in Italy, the level of educational attainment is not positively related to the probability of avoiding unemployment for immigrants of both genders, because foreign qualifications may not be sufficiently recognized by the receiving country. For this reason, it appears to be very important to promote a culture of integration versus the countries of origin of the main immigrants living in Italy.

If we analyze results concerning jobs requiring contacts with customers (*front office*), the associated coefficient is negative and significant for first-generation immigrants (but not for second-generation ones) meaning that for this kind of jobs, discrimination is mainly devoted to first-generation immigrants, most probably for statistical reasons (gaps in language knowledge and in professional skills).

Being able to develop effective strategies against ethnic discrimination presupposes a detailed analysis of the different types of discrimination (statistical or taste-based) and the subjects involved. In fact, policy implications are completely different depending on which of the two kinds of discrimination prevails. In the case of statistical discrimination, the solution in terms of policy would be to reduce asymmetric information (e.g., improving knowledge about foreign skills, education, and institutions), while in the case of taste-based discrimination, it would be to increase the culture of integration (e.g., intervention in schools and voluntary associations).

Despite the empirical evidence of the difference in treatment between natives and immigrants in the Italian labor market, Italy has not either a coherent immigration policy or an inclusive insertion policy (Schierup et al. 2006). Indeed, the main interest of policymakers appears to be driven by political interests rather than economical ones. For this reason, they are more concentrated on trying to avoid unauthorized entries than on the integration of immigrants (Zincone 2006). As also highlighted by Piazzalunga (2015), even though Italian and immigrant workers have the same social rights, no national policy tries to promote their economic and social integration.

## Footnotes

1 Search engines do not make any selection of the candidates, acting just as a repository for the CVs. Moreover, in several of the job postings analyzed, there is no information on the single firm offering vacancies because the search engine hid this information.

2 In the last years, we are experiencing a sort of “Islamophobia” in Europe—discrimination towards Muslim people increased following September 11th, 2001 events (Sheridan 2006)—and has risen even more in the very last years, due to terrorist activities conducted by the Islamic State of Iraq and the Levant (ISIL), often also referred to as “ISIS,” and the consequent shift in people’s view of Islam (Pargeter 2015; Fujimura 2016). A test conducted in France indicated that Muslim candidates are 2.5 times less likely to receive a callback than their Christian counterparts (Adida et al. 2010). The presence of Moroccans in our study can reveal whether being part of a Muslim country leads to a stronger discrimination.
